# Near-Infrared Fluorescence Imaging of Breast Cancer and Axillary Lymph Nodes After Intravenous Injection of Free Indocyanine Green

**DOI:** 10.3389/fonc.2021.602906

**Published:** 2021-03-09

**Authors:** Pierre Bourgeois, Isabelle Veys, Danielle Noterman, Filip De Neubourg, Marie Chintinne, Sophie Vankerckhove, Jean-Marie Nogaret

**Affiliations:** ^1^ Nuclear Medicine Service, Institut Jules Bordet, Université Libre de Bruxelles, Brussels, Belgium; ^2^ Surgery Service, Institut Jules Bordet, Université Libre de Bruxelles, Brussels, Belgium; ^3^ Department of Anatomo-Pathology, Institut Jules Bordet, Université Libre de Bruxelles, Brussels, Belgium

**Keywords:** breast cancer, axillary lymph nodes, indocyanine green, fluorescence imaging, sentinel lymph nodes

## Abstract

**Background:**

Near-infrared fluorescence imaging (NIRFI) of breast cancer (BC) after the intravenous (IV) injection of free indocyanine green (fICG) has been reported to be feasible. However, some questions remained unclarified.

**Objective:**

To evaluate the distribution of fICG in BC and the axillary lymph nodes (LNs) of women undergoing surgery with complete axillary LN dissection (CALND) and/or selective lymphadenectomy (SLN) of sentinel LNs (NCT no. 01993576 and NCT no. 02027818).

**Methods:**

An intravenous injection of fICG (0.25 mg/kg) was administered to one series of 20 women undergoing treatment with mastectomy, the day before surgery in 5 (group 1) and immediately before surgery in 15 (group 2: tumor localization, 25; and pN+ CALND, 4) as well as to another series of 20 women undergoing treatment with tumorectomy (group 3). A dedicated NIR camera was used for *ex vivo* fluorescence imaging of the 45 BC lesions and the LNs.

**Results:**

In group 1, two of the four BC lesions and one large pN+ LN exhibited fluorescence. In contrast, 24 of the 25 tumors in group 2 and all of the tumors in group 3 were fluorescent. The sentinel LNs were all fluorescent, as well as some of the LNs in all CALND specimens. Metastatic cells were found in the fluorescent LNs of the pN+ cases. Fluorescent BC lesions could be identified *ex vivo* on the surface of the lumpectomy specimen in 14 of 19 cases.

**Conclusions:**

When fICG is injected intravenously just before surgery, BC can be detected using NIRFI with high sensitivity, with metastatic axillary LNs also showing fluorescence. Such a technical approach seems promising in the management of BC and merits further investigation.

## Introduction

Indocyanine green (ICG) is a fluorescent cyanine dye used in medical diagnostics and approved by the Food and Drugs Administration and European Medicines Agency for several indications (1). Using near-infrared fluorescence imaging (NIRFI)-dedicated cameras, the intravenous (IV) injection of ICG enables imaging of the vascularization of the eye and transplants (2, 3). More recently, its use after intradermal and/or subcutaneous injection has been emphasized for lymphatic imaging in the evaluation of lymphedema (4, 5) and the detection of sentinel lymph nodes (LNs) (6–15). The ability of ICG to reveal various tumors after IV injection had also been demonstrated in both animals and humans (16–23).

In breast cancers (BC), its potential to enable their imaging in human patients was recognized as early as 2000 (24). Different subsequent studies (25–30) have shown that the kinetics of the accumulation and clearance of free ICG (fICG) allow the mammary cancerous tissues to be differentiated from healthy tissues. However, these data were obtained from a limited number of patients and at varying time points after fICG injection.

Because cancerous cells are found by pathologists near or in the surgical margins in 5.6 to 66% of cases after conservative surgery (31), a technical approach that would allow the visualization of tumor tissues and identification of tumor remnants after lumpectomy is of major interest. Currently, cryosection analysis represents the reference technique, but various techniques have been proposed for the evaluation and identification of tumor margins (32). Preliminary data of intraoperative NIRFI after the IV injection of methylene blue (MB) (33) or fluorescent molecules such as bevacizumab-IRDye800CW (34–39), among others, have been published. The injection of MB represents a simple approach but carries the risk of an allergic reaction (40), and fluorescent molecules such as bevacizumab-IRDye800CW present the main drawbacks of imaging agents in development, with all the limitations of such products.

This situation led us to launch the present studies in 2013 to evaluate the following in women undergoing surgery for histologically proven BC: 1) the best timing for fICG to be injected (the day before or just before the operation); 2) the accumulation of intravenously injected fICG in mammary tumors and in their respective axillary LNs; 3) the sensitivity of the approach in identifying these malignant lesions; and the potential contributions of such imaging methods in patients undergoing lumpectomy.

## Material and Methods

### Patients

The first study was approved by the Investigational Review Board (IRB) of the Jules Bordet Institute (CE2075) and was registered at ClinicalTrial.gov (NCT no. 01993576; https://clinicaltrials.gov/ct2/home) and the European Clinical Trials Database (EudraCT number 2013-000100-41; http://eudract.emea.europa.eu/). Between May 2013 and April 2014, twenty women (mean age, 60.3 years; range, 32 to 89 years) who were scheduled to undergo mastectomy (n = 19) or lumpectomy (n = 1) with selective lymphadenectomy (SLN) of axillary sentinel LNs (n = 5) and/or complete axillary LN dissection (CALND) (n = 15) for a histologically proven mammary tumor were enrolled in the study after providing written informed consent (see **Table S1** and **Table S2** for their characteristics). One patient (no. 8) was enrolled after neoadjuvant hormone therapy, and two (nos. 16 and 17) were enrolled in the framework of a relapse at the site of a previous lumpectomy.

The second study was also approved by the IRB of the Jules Bordet Institute (CE2200) and was registered at the ClinicalTrial.gov (NCT no. 02027818; https://clinicaltrials.gov/ct2/home) and the European Clinical Trials Database (EudraCT number 2013-005178-23; http://eudract.emea.europa.eu/). Between February and June 2014, twenty women (mean age, 60.1 years; range, 37 to 81 years) who were scheduled to undergo a lumpectomy with selective lymphadenectomy of axillary sentinel SLNs for a histologically proven mammary tumor were enrolled in the study after providing written informed consent (group 3: see **Table S3** for their characteristics).

The exclusion criteria were pregnancy, significant renal failure (creatinine >400 μmol/L), severe cardiac or pulmonary disease (ASA III-IV), a history of iodine allergy or anaphylactic reactions to insect bites or medication, and the presence or a history of hyperthyroidism. Patients were not limited in their normal behavior, diet, or medication intake before the study.

### Surgery and Specimen Preparation

A total of 0.25 mg of fICG/kg patient weight was intravenously injected in the first five patients (nos. 1–5) the day before the operation (group 1). Because fluorescence was detected in only two of the four tumors, the subsequent 15 patients (group 2) of our first study and every patient of our 2^nd^ study were injected immediately after anesthesia and before surgery.

The surgeons performed mastectomy or lumpectomy as usual, either preceded by the selective lymphadenectomy of the axillary sentinel LNs demonstrated by the pre-operative intra-mammary and peri-tumoral injections of radio-colloids (SLN; n = 25 patients) or followed by CALND (n = 15 patients). The delay between ICG injection and SLN dissection was 12–25 min, and that between ICG injection and CALND was 55–120 min.

**Figure 1:**
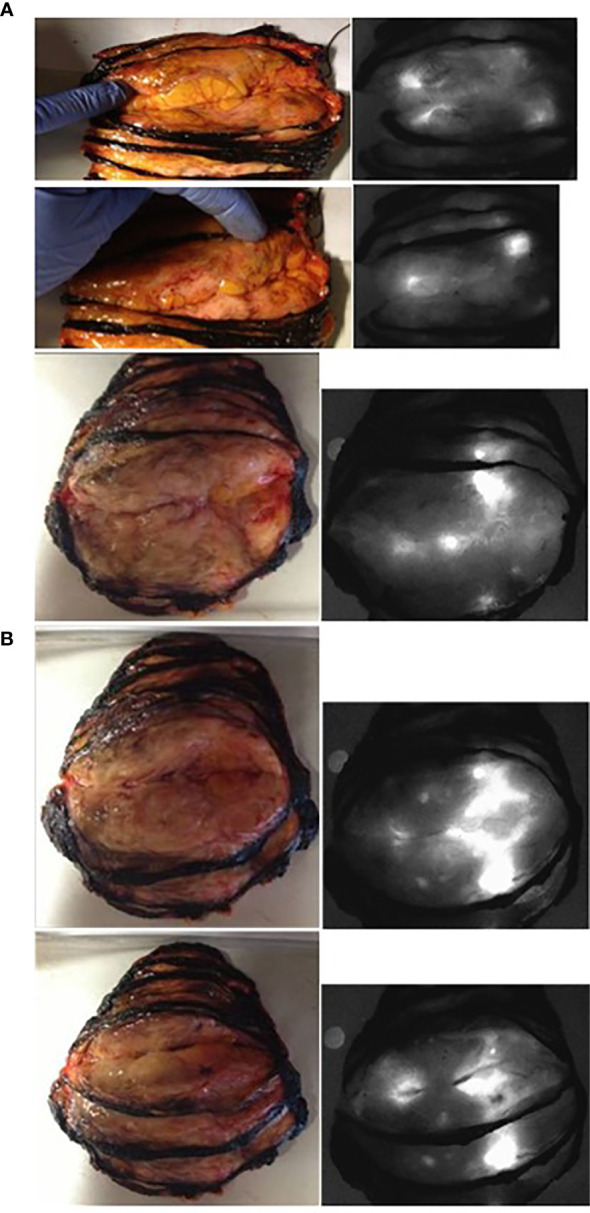
Normal and fluorescent images of the freshly sliced specimen from patient n° 20 in group 2 (panel **A**: the two ductal lesions—shown by the author’s finger—are fluorescent: the smallest was only 3 mm large) and from patient n° 19 in group 2 (panel **B**: with one lobular invasive).

**Figure 2:**
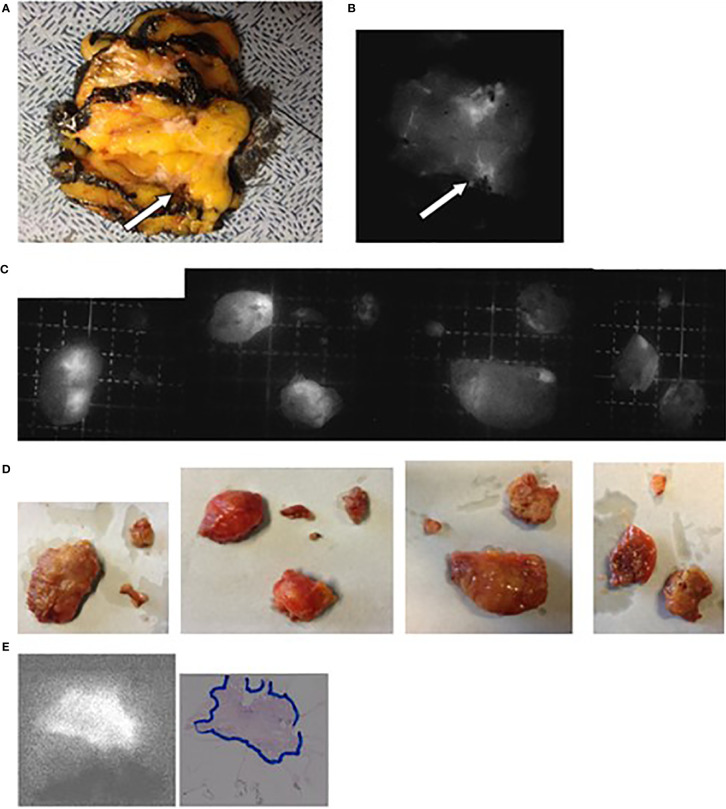
Images obtained for patient N° 8 who underwent a tumorectomy with CALND after neoadjuvant hormone therapy. **(A)** Lesion (arrow) on the freshly sliced specimen. **(B)** NIR fluorescence image of the same slices with the arrow showing the tumor. **(C, D)** Fluorescent and optical images of the axillary LNs. **(E)** Fluorescent images of the tumor embedded in paraffin and the corresponding H&E-stained pathological slices with delineation of the tumor tissues.

**Figure 3:**
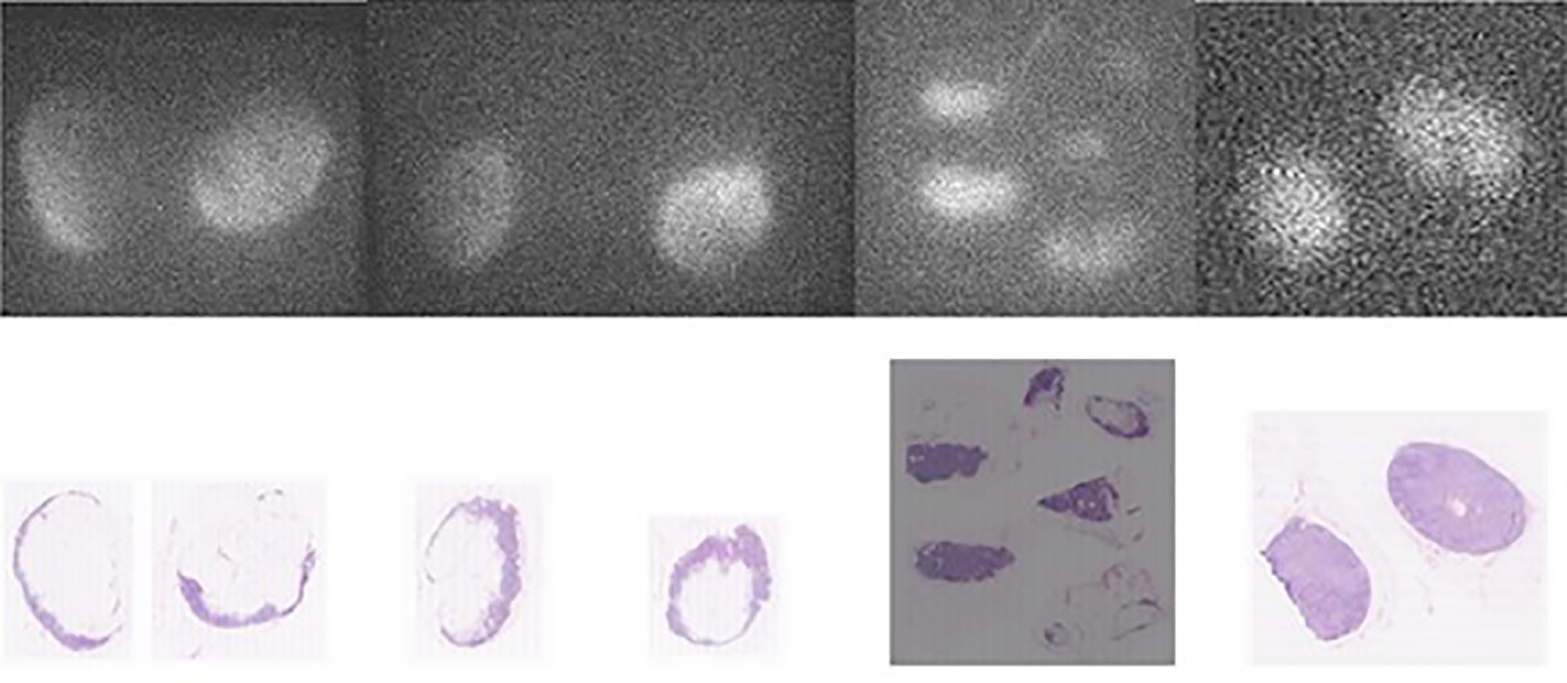
Comparison between fluorescence images of the lymph node embedded in paraffin (upper pictures) and the corresponding AP slices (lower pictures) obtained from pN- patients. From left to right, the first three sets of pictures correspond to lymph nodes from patient n° 8 (of group 2) and the last right-sided set of pictures are lymph nodes from patient n° 9 (of group 2).

The fresh specimens were always thereafter processed by the pathologist as usual. After dying the resection margins with India ink, each mastectomy and lumpectomy specimen was sliced at a thickness of 5–7 mm (see **Figure 1**) and 2–3 mm (see **Figure 2**), respectively, before being immersed in 4% buffered formalin overnight for fixation. For the mastectomy specimens, the area of interest, the tumor, and other areas after gross examination were also sliced thinner and processed as usual (dehydration and paraffin-embedding). Each LN was sliced at a thickness of 2 mm and fixed overnight in 4% buffered formalin before dehydration and paraffin-embedding.

### Fluorescence Imaging

**Figure 4:**
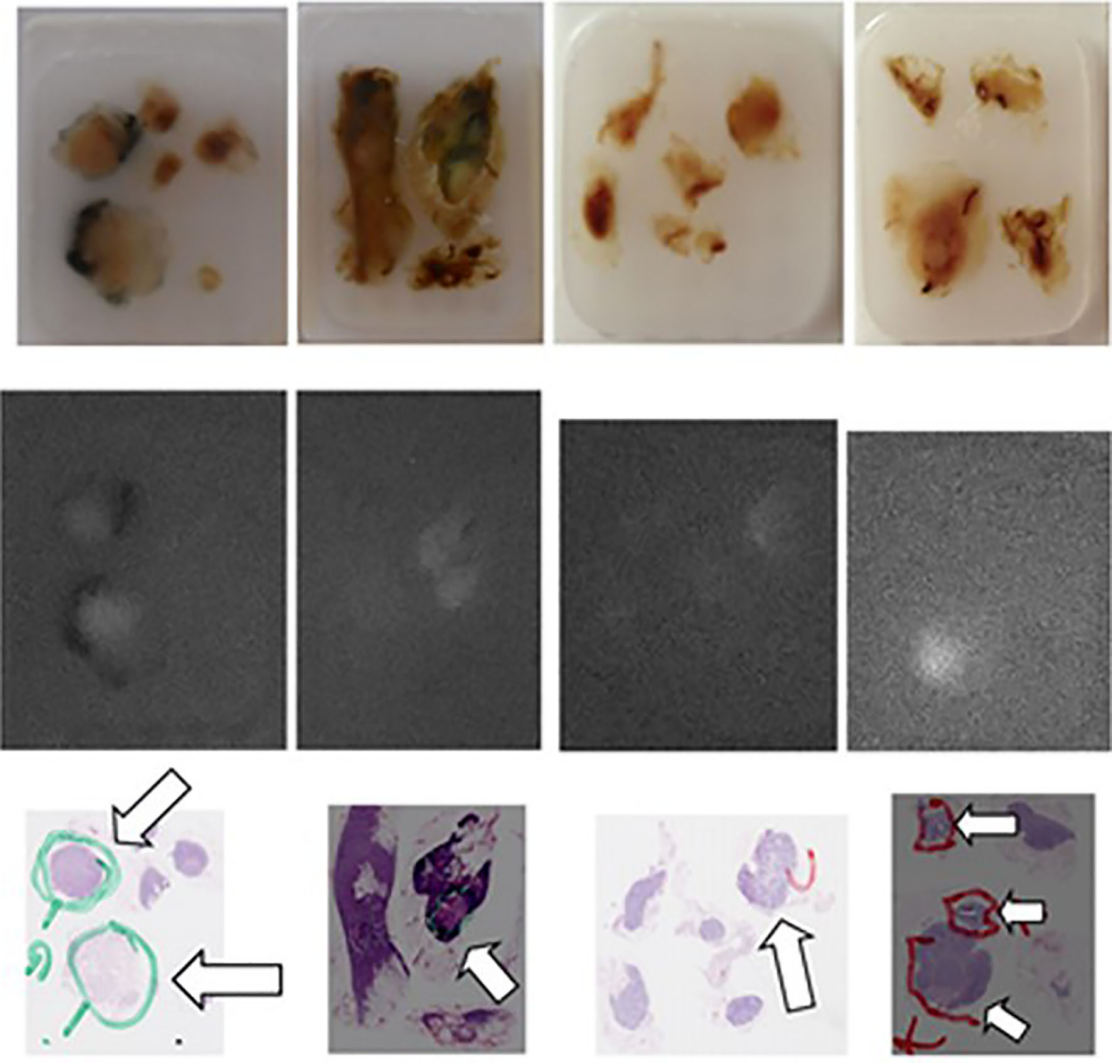
Comparison of the “real” images (upper panel), of the fluorescence images (mid panel) and of the corresponding AP slices of pN+ lymph nodes (arrows) embedded in paraffin obtained, from left to right, in patient n° 11 (two first series), in patient n° 18 (third series), and in patient n° 19 (fourth series) from Group 2.

Fluorescence images of the freshly sliced specimens (see **Figures 1A, B**), specimens after incubation in formalin (see **Figures 2A, B**), mammary lesions isolated by the pathologists, and all sentinel LNs and/or non-sentinel LNs before (see **Figures 2C, D**) and after embedding in paraffin (see **Figures 3** and **4**) were obtained under standard conditions in the department of anatomo-pathology using a dedicated NIR camera system (Photodynamic Eye, PDE; Hamamatsu Photonics, Hamamatsu, Japan).

**Figure 5:**
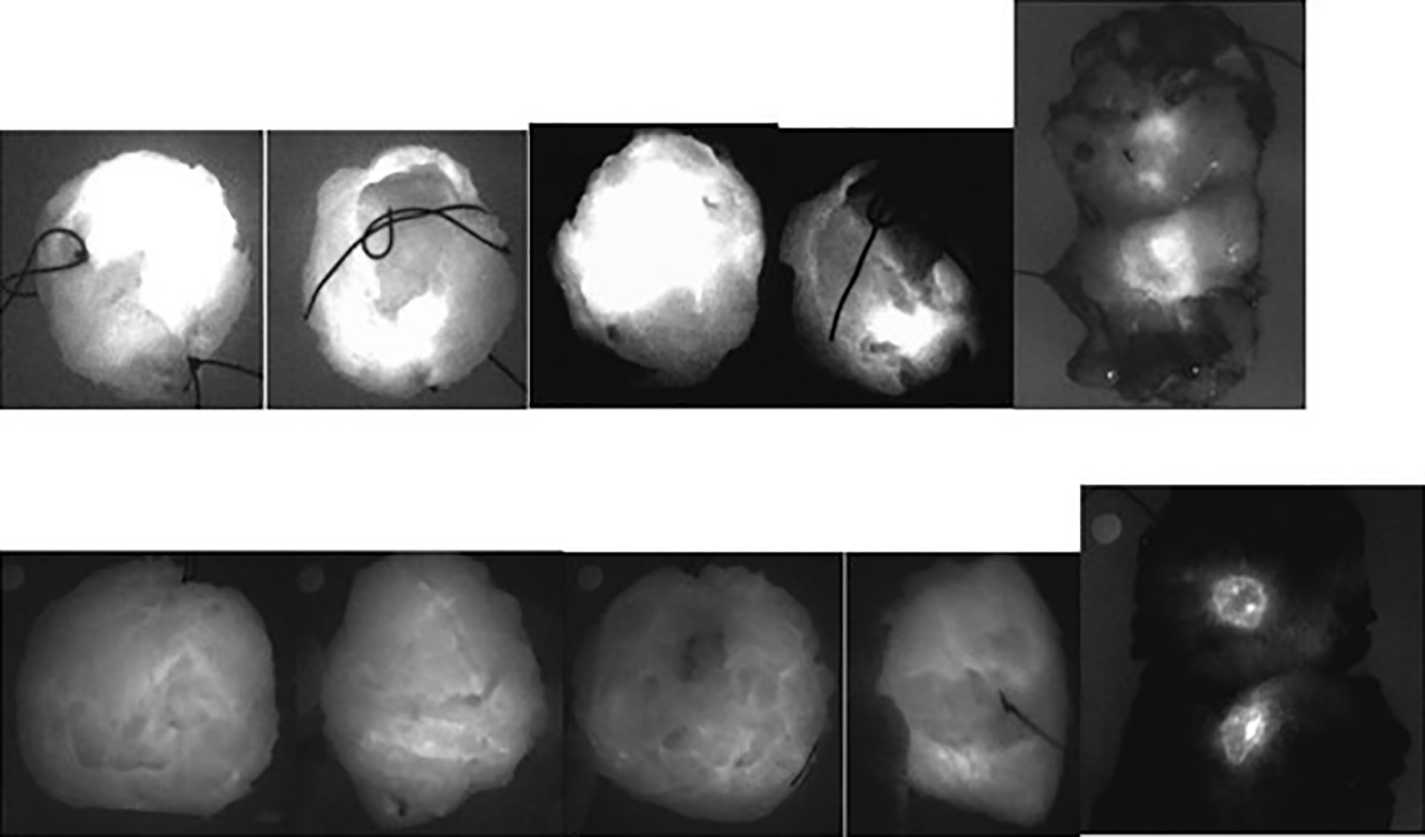
*Ex vivo* NIR fluorescence imagings of lumpectomy specimens (in the operating room) and of their slicings (in the department of Pathology) obtained: Upper panel: in patient n° 5 of Group 3 with a lobular invasive carcinoma, 19 × 10 × 11 mm large, close to the posterior margin (<1 mm) and to the anterior margin (4 mm) within a tumorectomy specimen sized 35 mm × 27 mm × 20 mm, histologically graded 1, with a maximum tumor to background fluorescence ratio equal to 2.0: the corresponding views of the whole specimen showed clear fluorescence at the surface of the specimen- Lower panel: in patient n° 6 of Group 3 with a canalar invasive carcinoma, 14 mm large within a tumorectomy specimen sized 62 mm × 75 mm × 35 mm, histologically graded 2 and with a maximum tumor to background fluorescence ratio equal to 4.0: the tumor was well centered and the corresponding views of the whole specimen showed no clear fluorescence at the surface of the specimen.

Fresh lumpectomy specimens were also imaged in the operating room (see **Figure 5**) using a dedicated NIR camera system (Photodynamic Eye, PDE; Hamamatsu Photonics, Hamamatsu, Japan).

### Semiquantitative Analysis of Fluorescence Images

Videos were recorded and converted to AVI format still images for semiquantitative image analysis. Based on white-light images, regions of interest (ROIs) were drawn over the tumor tissues and the adjacent healthy mammary tissue (considered “background”), and the fluorescence intensity (expressed in arbitrary units, AU) was measured using IC-CALC software. Finally, the tumor-to-background fluorescence ratio (fTBR) was calculated for each tumor at different steps: when fresh, after fixation in formalin, and after embedding in paraffin. Dissected LNs were processed the same way.

## Results

### Fluorescence of Mammary Tumors in Mastectomy and Lumpectomy Specimens

The first five patients in our 1st study were injected the day before surgery (group 1). In patient no. 1, the mammary lesion (one large *in situ* lesion limited to one-half of the breast) appeared faint but definitely more fluorescent than the normal mammary tissue. The largest LN, which was also fluorescent, was invaded by one ductal carcinoma. In the next four patients, who were also injected the day before surgery, only two mammary tumors fluoresced (**Table 1**).

**Table 1:** Fluorescence or not (and maximum fTBR) of tumors found in patients.

		Patient	Inj ICG	BC1 fluo?	BC2 fluo?	BC3 fluo?	fTBR
		n°.					
**1st study**	**Group 1**						
		1	D-1	(Yes)			(2.0)
		2	D-1	No			Neg
		3	D-1	Yes	Data lost		Data lost
		4	D-1	No			Neg
		5	D-1	Yes			3.5
	**Group 2**						
		6	Preop	Yes	Yes		5.0
		7	Preop	Yes	Yes		2.5
		8	Preop	Yes			3.0
		9	Preop	Yes	Yes		3.0
		10	Preop	Yes			ND
		11	Preop	Yes	Yes	Yes	4.0
		12	Preop	Yes			4.0
		13	Preop	Yes			2.5
		14	Preop	Yes	Yes		5.0
		15	Preop	Yes			4.5
		16	Preop	Yes			2.5
		17	Preop	Yes			2.0
		18	Preop	Yes	**No**		2.5
		19	Preop	Yes	Yes	Yes	3.0
		20	Preop	Yes	Yes		4.5
**2nd study**	**Group 3**						
		1	Preop	Yes			3
		2	Preop	Yes			2
		3	Preop	Yes			2
		4	Preop	Yes			3
		5	Preop	Yes			2
		6	Preop	Yes			4
		7	Preop	Yes			4
		8	Preop	Yes			1.7
		9	Preop	Yes			2
		10	Preop	Yes			2
		11	Preop	Yes			1.5
		12	Preop	Yes			2
		13	Preop	Yes			4
		14	Preop	Yes			2
		15	Preop	Yes			4
		16	Preop	Yes			1.5
		17	Preop	Yes			2.5
		18	Preop	Yes			2
		19	Preop	Yes			2.5
		20	Preop	Yes			3

(Inj ICG D-1, patients injected the day before surgery; Inj ICG Preop, patients injected immediately after anesthesia and before surgery.

In the subsequent five patients, who received an ICG injection immediately before the operation (following anesthesia), fluorescent mammary lesions were observed (**Table 1**). Thus, the last 10 patients were also injected immediately after anesthesia and before the operation.

In the 15 patients in group 2 who were injected just after anesthesia and before surgery (**Table 1**), 24 of the 25 clinically identified invasive cancerous lesions (14 ductal, 9 lobular, and 1 mucinous) were fluorescent, yielding a sensitivity of 96% in the mastectomy group. One tumor was not visualized well when examined as a fresh specimen (patient 18), and this was considered a false negative for fluorescence imaging because the lesion had been diagnosed on a biopsy performed the day before the mastectomy. No benign lesions were found in the mastectomy specimens and specificity of such ICG accumulation could not be established.

With regard to the possible influence of tumor size, one focus as small as 2 mm was detected in the multifocal lesion of one patient (no. 11), and small groups of cancerous cells dispersed on one surface 6 mm in diameter on the slide analyzed by the pathologist were reported in patient 17, who had mammary relapse.

Histological validation of ICG-positive tumors revealed a clear overlap between the fluorescence signals and tumor tissue (**Figure 2E**). In the last patient (no. 20), one tumor site was fluorescent, but no residual tumor was found after microscopic analysis. However, granulation tissue and inflammatory cells were observed.

When the lumpectomy specimens were imaged in the department of pathology after slicing, all the tumors were fluorescent, with no difference among the histopathological subtypes of tumors, giving an overall sensitivity of 100% in the lumpectomy group.

The mean maximum fTBR for the tumors (see **Table 1**) was 3.43 (standard deviation, ± 0.9) in the mastectomy specimens and 2.54 in the lumpectomy specimens (standard deviation, +/-0.73), and this metric did not seem to be influenced by the size (pT) of the tumors. However, this mean ratio in the lumpectomy group was significantly higher (2p < 0.05 using the Kruskal-Wallis test) for the 12 patients (of Group 3) with histological grade 2 or 3 tumors (2.83 +/− 0.67; range = 2.0–4.0, with four cases of fTBR = 2.0) than for the eight patients with grade 1 tumors (1.84 +/− 0.2; range = 1.5–2.0).

### Perioperative *Ex Vivo* Detection of Tumor Fluorescence in Lumpectomy Specimens

Tumor fluorescence was detected *ex vivo* in the operating room by NIR imaging of the whole lumpectomy specimens in 14 out of the 19 patients who were evaluated (Group 3: see **Figure 4**).

Tumor fluorescence could be detected in eleven out of thirteen lumpectomy specimens when the tumor size was larger than 10 mm but only three out of six lumpectomy specimens when the tumor size was less than 10 mm.

Among the 11 patients for whom we had the distance between the “closest” margins (n° 1, 4, 5, 7, 8, 10, 13, and 16–20: patient n° 8 was not evaluated in the operating room) and those for whom at least one surface of the tumor was fluorescent *ex vivo* (two surfaces were fluorescent for n° 5, 13, and 16), the tumor, except in one (patient n° 10), was not deeper than 5 mm (“final distance from invasive tumor to margin”) under (one of) the corresponding *ex vivo* fluorescent walls of the specimen. In other words, if the margin was defined as “close” when the tumor was within 5 mm from the resection margin, the corresponding surface was fluorescent in the operating room in 100% out of 10 patients, but fluorescence was observed in only one (patient n° 10) out of the three in whom the distance from the tumor to the margin was greater than 5 mm.

### *Ex Vivo* Free Indocyanine Green-FI of Axillary LNs

#### Patients Treated With Selective Lymphadenectomy

All the sentinel LNs were fluorescent in the 20 patients of Group 3 and in the four patients of Group 2 who underwent selective SLN guided by the preoperative peritumoral injection of radiocolloids.

#### Patients Treated With Complete Axillary Lymph Node Dissection

Among the 11 patients in group 2 who underwent CALND (**Table 2**), at least one of the dissected LNs was fluorescent in each patient. If the whole series is considered, a mean of 4.54 LNs per patient (from 1 to 8) were fluorescent, or 22.2% of all LNs found by the pathologist (from 10 to 60% if the number of fluorescent LNs is analyzed per patient).

**Table 2:** Number of LNs found by the pathologist in axillary specimens from patients in group 2.

Patient N°	Total LNs	pN+ LNs	Fluo LNs	pN + fluo LNs
6	16	0	2	0
7	22	0	5	0
8	34	0	8	0
9	7	0	4	0
11	35	4	7	4
14	21	3	5	3
15	29	0	5	0
16	10	0	6	0
18	9	1	1	1
19	13	5	4	2
20	29	0	3	0

total number, number of fluorescent LNs (fluo), pathologically positive LNs (pN+) and fluorescent and pN+ LNs (pN+ fluo).

The fluorescence in the normal LNs was localized and/or diffuse (**Figures 2C**, **3**, and **4**), sometimes with a (small or large) center that was not fluorescent (**Figure 3**). Histological validation of ICG-positive LNs showed a clear overlap between fluorescence and normal nodal tissues (**Figure 3**), with the hilum of the LNs not showing fluorescence.

In the four patients in group 2 who presented with LN metastases, 13 LNs harbored metastases, 10 (77%) of which were fluorescent. On the other hand, 17 (22%) of the 78 lymph nodes were fluorescent, and 10 (82%) of these 17 fluorescent LNs harbored metastases. In contrast, metastases were found in only 3 of the 61 non-fluorescent LNs, representing a false-negative rate (FNR) of 5%. The distribution of ICG in the metastatic LNs appeared somewhat different from what was observed in the normal LNs and in metastatic tumor areas.

## Discussion

### Indocyanine Green in Mammary Tumors

After IV injection, unbound fICG demonstrates rapid clearance from the blood circulation through the hepatobiliary system, with a half-life of 150 to 180 s (41). Because ICG has both lipophilic and hydrophilic properties, it also exhibits reversible binding to albumin and serum globulins, such as alpha1-lipoproteins. Unlike fICG, the complex formed by ICG and these proteins behaves like a macromolecule in the circulation.

With regard to our field of application in patients with tumors, the imaging of tumors immediately after the injection of ICG depends on their relative hypervascularization. In 2008, Wall et al. (42) demonstrated the correlation between the intensity of fluorescence and tumor vascularization in mammary tumor graft-bearing animals (MCF7 cell line).

Cancer tissues also exhibit “vascular fenestrations,” which allow the extravasation of molecules over 50 kDa in molecular weight (MW) (43). In contrast, normal tissues have smaller vascular fenestrations that prevent the extravasation of molecules over 20 kDa in MW (44).

Two mechanisms, hypervascularization and the enhanced permeability retention (EPR) effect, can thus explain the ICG fluorescence of mammary tumors, but theoretically with potential differences in terms of sensitivity (and of specificity) over time.

Our observation that the mean maximum fTBR was higher in histological grade 2 and 3 tumors than in grade 1 tumors supports the hypothesis of fluorescence related to the EPR effect. Daldrup et al. (45) showed in mammary tumor-bearing animals that capillary hyperpermeability to proteins with a high MW (i.e., the EPR effect) increases with the histological grade of tumors.

### Sensitivity and Specificity of Indocyanine Green Near-Infrared Fluorescence Imaging for the Detection of Mammary Tumor Tissues

In the present series (with the injection of 0.25 mg per kg of patient weight), the accumulation of ICG as observed *ex vivo* in sliced specimens appears highly sensitive, with 44 of the 45 tumors showing fluorescence. The “falsely negative” non-fluorescent tumor had been biopsied the day before surgery, and the hematoma observed on pathology could explain its negative fluorescence. However, no benign lesions were found in the mastectomy specimens, and the specificity of such ICG accumulation could not be evaluated in our study.

Using *ex vivo* optical breast imaging after the IV injection of fICG (25 mg as a bolus), Schneider et al. (29) reported a sensitivity of 85.7% based on the positive detection of ***in vivo*** fluorescence in 12 out of 14 malignant lesions and a specificity of 87.5%, with no *in vivo* fluorescence detected in seven of eight benign lesions. A false-negative result was found in one patient for a micropapillary carcinoma (although the pT was 16 mm) and in another patient due to the presence of necrotic tissue at the center of the lesion.

Using intraoperative NIR imaging in 12 patients with BC but with fICG being injected 24 h before the operation and at a relatively high concentration (5 mg/kg), Keating et al. (46) reported fluorescence in all tumors, while in the department of pathology, we identified fluorescence in only two out of four tumor specimens when ICG was injected the day before the operation at a concentration of 0.25 mg/kg.

In the article published recently by our group (47), the sensitivity of ICG (injection unchanged) for detecting the fluorescent tumor in lumpectomy specimen was lower (31/35 or 87%). However, this result was obtained with a different NIRFI camera-device (and other parameters) and this difference stresses the importance of defining the optimal acquisition parameters for each imaging system. Interestingly, the false negatives were four of the (32) ductal carcinomas, which also showed the lowest fTBR in two of our patients with ductal cancer.

### Timing of Imaging After IV Indocyanine Green Injection

Not only the amount of ICG injected but also the timing of the injection thus appears to influence the fluorescence of mammary tumors. In addition, when the studies were initiated, the data were somewhat confusing. In animals, Reynold et al. (19) reported that spontaneous mammary tumors (in two female dogs) remained fluorescent for up to 120 min after the injection of ICG (which corresponds to the “vascular” phase of the tracer), and Gurfinkel et al. (18) found that, in one female dog operated on for a spontaneous mammary tumor, fluorescence remained detectable for 72 h after ICG injection (which is in agreement with the hypothesis of the extravasation and retention of ICG-labeled proteins). In patients and on ***ex vivo*** optical breast imaging after the IV injection of fICG (25 mg as a bolus), Poellinger et al. (30) reported the results of an analysis by two different readers of images recorded for during the IV administration of ICG (early imaging) and approximately 25 min after the IV injection of ICG (delayed imaging). The sensitivity of early imaging was low (~50 and 67%), but the corresponding specificity was high (88 and 75%). The sensitivity of delayed imaging was 85 and 92%, and the corresponding specificity was 75 and 62%. A higher mean contrast value was also reported for delayed imaging than for early imaging (0.64 *vs.* 0.25).

### Potential Clinical Applications of Free Indocyanine Green Fluorescence Imaging in Breast Cancer

Although fICG is not tumor-specific, our data show that when ICG is injected at a concentration of 0.25 mg/kg just before the operation, it allows visualization of the tumor and not of the surrounding healthy tissues, which may have practical implications. For the purpose of intra- and postoperative diagnostic imaging, the identification of any abnormal tissues (sensitivity) is more important than specificity for tumor detection. Several new optical imaging agents targeting specific cell surface markers, such as HER2 receptor (48), have been reported, but these specific cellular markers may or may not be expressed on all cancer cells and are only related to tumors expressing these biomarkers on the cell surface (only 15–20% for HER2). Furthermore, these markers will have to be approved by the relevant authorities before use in clinical applications. Such agents could demonstrate superior sensitivity and specificity to ICG and merit consideration. However, none of these agents are as easy to apply in humans as ICG.

We identified four fields of clinical applications for the IV injection of fICG in the management of BC.

First, the detection of such fluorescence may be used by pathologists to look for the limits of mammary lesions and may represent an easy way to determine the tumor margin status. This approach could be particularly useful in cases where gross examination cannot allow clear mapping of the tumor limits (e.g., extensive fibrosis) and/or in cases of special patterns of infiltration without a fibrotic reaction of the stroma (e.g., lobular growth pattern with tumor cells infiltrating the tissue in single file or as single cells); these pathological situations can be anticipated on the basis of biopsies and/or radiological investigations.

Second, the perioperative detection of fluorescent lesions while the surgeon is performing lumpectomy represents another possible application of such fluorescence imaging. When the surgeon has cut the skin above the tumor location, the fluorescence emitted by the tumor may be detected and used to guide lumpectomy. Keating et al. (46) reported that the IV injection of 5.0 mg/kg of fICG 24 h before the operation allowed the positive identification of all 12 patients’ breast tumors by intraoperative NIR imaging *in situ*; in the present study, with the injection of a lower concentration when the patient was anesthetized, we were able to observe tumor fluorescence in only 15 out of 19 cases ***ex vivo*** and mainly in the lumpectomy specimens of patients in which the tumor was close to the margin of the specimen. Our ability to visualize tumors is thus dependent on not only the concentration of ICG (injected and finally located in the tumor) but also the attenuation of the fluorescence by surrounding tissues.

This 2^nd^ result may represent an advantage but also a drawback with regard to the third field of application: the detection of remnants by imaging of the surgical margins. Here, we feel it is important to stress the work reported by Madajewski et al. (49). These authors demonstrated the ability of fICG to show the limits of tumor areas (grafts in animals derived from cells of pleural, breast, lung, and esophageal cancer) and more precisely to identify the presence of malignant fluorescent remnants in surgical margins previously considered negative at first glance by the surgeon. The survival in the group in which these fluorescent remnants were removed was better than the survival in the control group, in which no such additional ICG-guided resection was performed. In a similar approach, Jiang et al. (50) confirmed the interest in NIR-guided surgery and reported the results of a series of 60 mice bearing 4TI BC tumors in their flank that traditional margin assessment identified 30% of positive margins, while NIR imaging identified 90% of positive margins.

Using NIR imaging after the injection of ICG, the problem of tumor remnants can be approached in two complementary ways. First, the tumor specimen, once *ex vivo*, can be examined for fluorescence emitted by the tumor. In the present series, fluorescence emitted by the tumor could be detected *ex vivo* on the surface of the lumpectomy specimen in 14 of the 19 evaluated cases but in only three out of six with a diameter <11 mm and in 11 out of 13 with a diameter >10 mm. The fact that the fluorescent tumors were also frequently close to the margin (12 being reported by the pathologist with a distance from the tumor to the margin of the lumpectomy specimen of 5 mm or less) also explains the detection of tumor fluorescence and might represent the most important factor allowing detectability when observing fresh specimens *ex vivo*, even for small tumors (<10 mm). However, such an approach would only allow (at the present stage of our developments) fluorescent areas at risk (of being positive) to be observed *ex vivo* and to be controlled *in vivo*. The second approach is more direct and consists of controlling the surgical bed and searching for and removing any remaining fluorescent tissue.

The feasibility of such NIR imaging, perioperatively and in the framework of conservative surgery, to observe fluorescence from BC tissue (and remnants) was reported by Tummers et al. (33), but after the IV injection of MB, another NIR fluorophore. Their overall identification rate was 18/21 for invasive carcinomas. This slightly lower sensitivity than that of ICG (at least 96% in the present study) might be explained by the greater attenuation with MB (penetration depth = approximately 5 mm) than with ICG (penetration depth = around 10 mm). Another hypothetical explanation might be that the cationic structure of MB would render MB the substrate of multidrug-resistant proteins known to be present in several types of BC (51) and that MB would thus be cleared from the tumor in such cases, with no or low fluorescence. Finally, Tummers et al. also reported (33) that they did not detect one mucinous adenocarcinoma, whereas such a lesion was detected by ICG fluorescence in our first series of patients treated with mastectomy. Of utmost interest, they also detailed that, in two patients with pathologically positive resection margins, fluorescent tumor tissues could be identified perioperatively on the surface of the resected specimen and/or in the wound bed.

Keating et al. also found (46) that ICG fluorescence could be detected in six cases in the surgical bed following lumpectomy, although all their patients had clear margins. With the high concentration of ICG they administered (also injected the day before), the fluorescence seems to no longer be limited to the tumor (as in our series) but to diffuse into the surrounding healthy tissues (Keating et al. mention in the discussion of their article that smears of fluid from the specimen were fluorescent) (46), which thus limits the value and potential of their approach in the detection of tumor remnants.

In a previous study, we had reported that the negative predictive value was 100% for the *ex vivo* ICG-FI detection of viable BCs tissues after neoadjuvant chemotherapy (52) as well as for the *in vivo* detection of ICG fluorescence in the tumoral bed after lumpectomy (47). The major contribution of the technique seems thus to be its excellent NPV, allowing the surgeon confidence that resection margins are clean if no residual fluorescence is visualized in the tumoral bed.

### Fluorescence Imaging of Axillary Lymph Nodess After IV Indocyanine Green Injection

When our study was launched, the systematic presence of ICG fluorescence in the sentinel LNs and some LNs collected by CALND was unexpected despite some preliminary observations in the literature. Reynold et al. (19) reported in 1999 that the IV injection of ICG allowed the detection of fluorescence in draining reactive LNs of spontaneous mammary tumors in two female dogs and that these structures remained fluorescent for up to 120 min after the injection of ICG. In humans, such visualization of fluorescence in LNs after the IV injection of fICG was reported in 2013 by Yokoyama et al. in patients who underwent lymphadenectomy for the nodal relapse of head and neck cancer (53). Our group confirmed their observation in 2016 in a series of 11 patients who had undergone cervical lymphadenectomy for primary and relapsing head and neck cancer (54) but also reported such accumulation of ICG in metastatic LNs in cases of colorectal and ovarian cancer (55–57).

In our patients who underwent SLN after peri-tumoral injection of radio-colloids, fluorescence was observed in all sentinel LNs. Due to the short delay between the excision of the sentinel LNs and the injection of ICG (<15 min), the fluorescence may be related to vascularization.

In patients who underwent CALND 50 to 120 min after the IV injection of ICG, no definitive explanations can be proposed regarding why some LNs remained fluorescent. At this stage of our analysis, we can propose two explanatory hypotheses: one consists of ICG accumulation *via* metastatic tumor tissues for pN+ nodes, and the other consists of ICG accumulation by normal nodal tissues in an inflammatory state, stimulated by factors arising from the tumoral bed.

These results might be interesting for pathologists. The simple categorization of fluorescent LNs gives the following values (diagnosing one LN as pN+ on pathological examination): sensitivity and specificity of 77 and 81%, respectively, with a low positive predictive value of 20% but a high negative predictive value of 98.2%. In our limited series of 11 patients, pathologists have also isolated and analyzed 225 LNs but found only 13 pN+ LNs (in four patients) on histological examination, for an efficiency rate of only 5.5%; however, if they had isolated and analyzed the 50 fluorescent LNs, they would have found 10 pN+ LNs, for a higher efficiency rate of 20%. However, analysis of only the fluorescent LNs would have led to underestimation of the prognostic risk (related to the number of pN+ LNs) in one of the four pN+ patients, for whom only two of the pN+ LNs were fluorescent. The sensitivity of our ICG imaging (to show the lymph nodes at risk) may appear “low” and is based on *ex vivo* imaging. However, the aim of our study was not to evaluate the per-operative detection of fluorescent sentinel lymph nodes after the IV injection of ICG and we think that the lymphatic approaches after sub-cutaneous and/or intra-mammary injection of a dye and/or radio-tracer will remain the standard methods. HowH

These observations also concern a limited number of pN+ patients, and the next challenge will be to determine whether this technique can be used more specifically by pathologists to detect LNs at risk and with metastatic deposits without a loss of sensitivity or prognostic value.

## Conclusion

Injecting fICG at a concentration of 0.25 mg/kg just before surgery seems to be an approach of interest in the management of patients with BC. In all, 44 out of 45 tumors were fluorescent. Fluorescent tumor tissues could also be identified perioperatively on the surface of the resected lumpectomy specimen in 14 of 19 cases. The fluorescence depended on the depth and location of the tumor in the specimen and on the histological grade of the tumor. The approach might be of interest in the management of patients with BC in order to perioperatively observe fluorescent tumor tissue and/or, more importantly, the persistent fluorescence of tumor remnants in the surgical bed. The unexpected detection of ICG fluorescence in some LNs obtained by CALND after IV injection of the dye and the presence of metastatic cells in these fluorescent LNs merit further investigations.

## Data Availability Statement

The raw data supporting the conclusions of this article will be made available by the authors, without undue reservation.

## Ethics Statement

The studies involving human participants were reviewed and approved by the Investigational Review Board (IRB) of the Jules Bordet Institute (CE2075 and CE2200), 1, rue Heger-Bordet, 1000, Brussels, Belgium. The patients/participants provided their written informed consent to participate in this study.

## Authors Contributions

PB, IV, and DN conceptualized the study. PB acquired the funding and developed the methodology. IV, DN, J-MN, FDN, MC, and PB provided the resources. PB and J-MN supervised the study. PB conducted the project administration. SV was in charge of the data curation. SV, MC, and PB conducted the investigation. PB prepared the original draft. J-MN, IV, DN, MC, and PB reviewed and edited the manuscript. All authors contributed to the article and approved the submitted version.

## Funding

The studies were supported by a grant from « Les Amis de l’Institut Bordet ». The funders played no role in the study design, data collection or analysis, decision to publish, or preparation of the manuscript.

## Conflict of Interest

The authors declare that the research was conducted in the absence of any commercial or financial relationships that could be construed as a potential conflict of interest.
