# Supplementary material for: Near-Infrared Fluorescence Imaging of Breast Cancer and Axillary Lymph Nodes After Intravenous Injection of Free Indocyanine Green
Source: Front Oncol. 2021 Mar 9;11:602906. doi: 10.3389/fonc.2021.602906 (PMC7985064; doi:10.3389/fonc.2021.602906)
Supplement: Supplementary file 1 [file DataSheet_1.pdf]

**Table S1: Demographic and individual clinical data of patients included in the first study** (M = mastectomy; T = tumorectomy; CALND = complete axillary lymph node dissection; SLN = sentinel lymph node-selective lymphadenectomy; Lat = lateralization, right or left; Menop = menopausal status)

| No. | Breast Surg | Ax Surg | Age | Lat | Menop | Clinical status |
|---|---|---|---|---|---|---|
| 1 | M | CALND | 64 | L | Post | cN1 |
| 2 | M | CALND | 74 | L | Post | Multifocal |
| 3 | M | CALND | 89 | R | Post | Multifocal cN1 |
| 4 | M | CALND | 38 | L | Pre | Multifocal |
| 5 | M | SLN | 58 | L | Post | Unifocal |
| 6 | M | CALND | 43 | R | Pre | Bifocal |
| 7 | M | CALND | 52 | R | NE | Bifocal |
| 8 | T | CALND | 71 | R | Post | Unifocal |
| 9 | M | CALND | 66 | R | Post | Bifocal |
| 10 | M | SLN | 55 | R | Pre | Bifocal |
| 11 | M | CALND | 68 | R | Post | Trifocal |
| 12 | M | SLN | 50 | R | Peri | Unifocal |
| 13 | M | SLN | 70 | R | Post | Unifocal |
| 14 | M | CALND | 57 | R | Post | Bifocal |
| 15 | M | CALND | 78 | R | Post | Unifocal |
| 16 | M | CALND | 32 | L | Pre | Relapse |
| 17 | M | SLN | 60 | R | Post | Relapse |
| 18 | M | CALND | 43 | R | Pre | Bifocal |
| 19 | M | CALND | 49 | R | Post | Trifocal |
| 20 | M | CALND | 65 | R | Post | Bifocal |

**Table S2: Anatomo-pathological characteristics of tumors in groups 1 and 2**

| Patient N° | Histology | pT (mm) | Final pTN | Grade | RO | RP | ki67 (%) | Neu |
|---|---|---|---|---|---|---|---|---|
| 1 | In situ (is) high grade | 55 (is) | TisN3 | ND | 0 | 0 | 30 | 3+ Fish+ |
| 2 | Ductal cribriform | 21, 19, 11, 5 | pT1cN0 | 1 | 8 | 7 | 5 | Neg |
| 3 | Ductal and lobular | 60-70, 8, 21 | pT3N0 | 1 | 8 | 8 | <10 | Neg |
| 4 | Ductal | 12 | pT1cN0 | 2 | 8 | 5 | 10 | Neg |
| 5 | Ductal | 18 | pT1cN0(sn) | 3 | 8 | 7 | ND | Neg |
| 6 | Ductal | 6, (3+2+1) | pT1bmN0 | 2 | 8 | 8 | 20 | 3+ Fish+ |
| 7 | Lobular | (10+6), 5 | pT1bN0 | 1 | 7 | 4 | <10 | 1+2+ Fish- |
| 8 | Mucinous | 18 | ypT1cN0 | 1 | 8 | 0 | <5 | Neg |
| 9 | Lobular | 11, 7 | pT1cmN0 | 3 | 8 | 8 | 30 | Neg |
| 10 | Ductal | 7 | pT1bN0(sn) | 1 | 8 | 7 | 10 | Neg |
| 11 | Ductal | 30, 13, 2 | pT2mN2a | 2 | 8 | 8 | <5 | Neg |
| 12 | Lobular | 18 | pT1cN0(sn) | 2 | 8 | 8 | 15 | Neg |
| 13 | Lobular | 8 | pT1bN0(sn) | 1 | 8 | 8 | 10 | Neg |
| 14 | Ductal | 35, 5 | pT2mN1a | 2 | 8 | 6 | 10 | Neg |
| 15 | Ductal | 21 | pT2N0 | 3 | 0 | 0 | 20 | 3+ Fish+ |
| 16 | Ductal | 5 | rpT1aN0 | 2 | 7 | 5 | 40 | 3+ Fish+ |
| 17 | Ductal | 6* | rpT1bN0(sn) | 2 | NC | 0 | 20 | 3+ Fish+ |
| 18 | Ductal | 10, 9 | pT1cmN0(i+) | 2 | 7 | 7 | 15 | Neg |
| 19 | Lobular | 22, 15, 10 | pT2mN2 | 2 | 8 | 6 | 10 | Neg |
| 20 | Ductal | 16, 3*/** | pT1cmN0 | 2 | 8 | 6 | 10 | Neg |

*(see text) ** size on biopsy

**Table S3: Demographics and clinical data of the patients in our 2nd study (group 3)** (M = mastectomy; T = tumorectomy; CALND = complete axillary lymph node dissection; SLN = sentinel lymph node-selective lymphadenectomy)

| Patient N° | Age (years) | AP | pT mm | pTN | Grade | RO | RP | ki67 | Her2 |
|---|---|---|---|---|---|---|---|---|---|
| 1 | 75 | Ductal | 25 | pT2N0snmi | 2 | 8 | 5 | 25 | Neg |
| 2 | 70 | Papillary | 7 | pT1bN0sn | 1 | 8 | 5 | 10 | Neg |
| 3 | 50 | Lobular | 9 | pT1bN0sn | 2 | 8 | 8 | <10 | Neg |
| 4 | 54 | Lobular | 26 | pT2N1snmi | 2 | 8 | 0 | <5 | Neg |
| 5 | 70 | Lobular | 19 | pT1cN0sn | 1 | 8 | 5 | <5 | Neg |
| 6 | 49 | Ductal | 14 | pT1cN0sn | 2 | 4 | 4 | 20 | Neg |
| 7 | 81 | Ductal | 15 | pT1cN1a | 3 | 3 | 0 | 15 | 3+ Fish + |
| 8 | 48 | Ductal | 12 | pT1cN0sn | 1 | 7 | 8 | <5 | Neg |
| 9 | 55 | Ductal | 16 | pT1cN0sn | 3 | 0 | 0 | 90 | Neg |
| 10 | 60 | Ductal | 5 | pT1aN0sn | 1 | 8 | 8 | 10 | Neg |
| 11 | 59 | Lobular | 15 | pT1cN0sn | 1 | 8 | 8 | 5 | Neg |
| 12 | 57 | Ductal | 9 | pT1bN0sn | 1 | 8 | 8 | <10 | Neg |
| 13 | 59 | Duc-Lob | 16 | pT1cN0sn(i+) | 2 | 8 | 7 | 25-40 | 2+ Fish + |
| 14 | 59 | Ductal | 11 | pT1CN0sn(i+) | 1 | 8 | 7 | <5 | Neg |
| 15 | 55 | Ductal | 15 | pT1cN0sn | 2 | 8 | 0 | <10 | 2+ Fish - |
| 16 | 71 | Ductal | 11 | pT1cN0sn(i+) | 1 | 8 | 8 | 10 | Neg |
| 17 | 70 | Ductal | 14 | PT1cNosn(i+) | 2 | 8 | 5 | 10 | 2+ Fish + |
| 18 | 69 | Ductal | 9 | pT1bN0sn | 2 | 8 | 8 | 10 | 2+ Fish - |
| 19 | 37 | Ductal | 10 | pT1cN0sn | 2 | 8 | 8 | 15 | Neg |
| 20 | 54 | Ductal | 11 | pT1cN0sn | 2 | 0 | 0 | 70 | Neg |
